# Toxicological assessment of *Phormidium sp.* derived copper oxide nanoparticles for its biomedical and environmental applications

**DOI:** 10.1038/s41598-023-33360-3

**Published:** 2023-04-17

**Authors:** Nida Asif, Rakhshan Ahmad, Samreen Fatima, Shehzadi Shehzadi, Tabassum Siddiqui, Almaz zaki, Tasneem Fatma

**Affiliations:** 1 Department of Biosciences, Jamia Millia Islamia, Jamia Nagar, New Delhi, 110025 India; 2 Department of Biotechnology, Jamia Millia Islamia, New Delhi, 110025 India

**Keywords:** Microbiology, Biomaterials, Nanoscale materials, Microbiology techniques, Microscopy, Nanobiotechnology

## Abstract

Driven by the need to biosynthesized alternate biomedical agents to prevent and treat infection, copper oxide nanoparticles (CuONPs) have surfaced as a promising avenue. Cyanobacteria-derived synthesis of CuONPs is of substantive interest as it offers an eco-friendly, cost-effective, and biocompatible route. In the present study biosynthesized CuONPs were characterized and investigated regarding their toxicity. Morphological analysis using TEM, SEM and AFM showed the spherical particle size of 20.7 nm with 96% copper that confirmed the purity of CuONPs. Biogenic CuONPs with IC_50_ value of 64.6 µg ml^−1^ showed 90% scavenging of free radicals in superoxide radical scavenging assay. CuONPs showed enhanced anti-inflammatory activity by 86% of protein denaturation with IC_50_ value of 89.9 µg ml^−1^. Biogenic CuONPs exhibited significant toxicity against bacterial strains with lowest MIC value of 62.5 µg ml^−1^ for *B. cereus* and fungal strain with a MIC value of 125 µg ml^−1^ for *C. albicans*. In addition CuONPs demonstrated a high degree of synergistic interaction when combined with standard drugs. CuONPs exhibited significant cytotoxicity against non-small cell lung cancer with an IC_50_ value of 100.8 µg ml^−1^ for A549 and 88.3 µg ml^−1^ for the H1299 cell line with apoptotic activities. Furthermore, biogenic CuONPs was evaluated for their photocatalytic degradation potential against methylene blue dye and were able to removed 94% dye in 90 min. Free radical scavenging analysis suggested that CuONPs assisted dye degradation was mainly induced by hydroxide radicals. Biogenic CuONPs appears as an eco-friendly and cost effective photocatalyst for the treatment of wastewater contaminated with synthetic dyes that poses threat to aquatic biota and human health. The present study highlighted the blend of biomedical and photocatalytic potential of *Phormidium* derived CuONPs as an attractive approach for future applications in nanomedicine and bioremediation.

## Introduction

Worldwide, use of nanoparticles (NPs) in the biomedical field attracted the attention of researchers. The small surface to volume ratio compared to its bulk material is responsible for their enhanced or unique properties. As it enable the NPs to interact with high degree of specificity and enhanced efficacy to combat infectious diseases^1,2^. Among the various metal oxides NPs copper oxide nanoparticles (CuONPs) gained significant interest due to their high stability, longer shelf life, high quantum efficiency and antimicrobial activity. Extensive biomedical application of CuONPs as an antioxidant, antimicrobial, anti-inflammatory, antiviral, cytotoxic and anticancer activities has made them strong candidates to be used as therapeutic agents^3–7^. Longer shelf life and stability also make CuONPs the suitable candidate in environmental biotechnology for purification of water and elimination of pollutants from industrial effluents without the formation of harmful by products^8^. In human body copper (Cu), is present in trace element that is found in enzymes such as superoxide dismutase, cytochrome oxidase, and tyrosinase^6^. Moreover it serves as a cofactor for multiple enzymes responsible in neuropeptide production, cell signalling mechanism, oxidative stress, and immune cell function in humans^9^.

Synthesis of CuONPs can be done by several physical, chemical and biological processes. Physical and chemical synthesis approaches suffers from drawbacks like expensive reagent, hazardous reaction condition, longer time, tedious process to isolate NPs and harm to ecosystems as well as human health^10,11^. Thus, to overcome these shortfalls, the principle of green chemistry utilizing naturally available resources like viruses, bacteria, cyanobacteria, fungi, algae and plants were utilized for the fabrication of NPs^12^. Cyanobacteria (blue–green algae) form one of the largest and most primitive ancestral groups with prokaryotic cell structures that have the capability of carbon dioxide-dependent photosynthesis. Cyanobacteria have received a lot of scientific attention for their ability to synthesize NPs, not only due to their high biomass efficiency, but also due to their potential to bioremediate hazardous metals and transform them to more manageable forms^13^. These are able to synthesize inorganic and metal oxide NPs e.g. selenium, zinc, platinum, palladium, gold, and silver nanoparticles^14–18^. Cyanobacteria facilitated synthesis of NPs occurs either through extracellularly, that involves the production of reductase enzyme due to electrostatic interactions and intracellularly i.e. within the cells by the activity of enzymes^13^.

To the best of our knowledge, studies on biomedical applications of CuONPs are limited, prompting us to undertake the present study, to unravel the toxicological evaluation of biologically synthesized CuONPs. In the present study CuONPs were biologically synthesized using cyanobacteria (*Phormidium sp*.) cell extract and characterized by Scanning electron microscopy (SEM), Transmission electron microscopy (TEM), energy dispersive x-ray spectrum (EDX), X-ray diffraction (XRD), Atomic force microscopy (AFM), Fourier transform infrared spectroscopy (FTIR) and UV–Vis analysis. As-synthesized CuONPs were studied for antioxidant, antimicrobial, and anti-inflammatory properties. Cytotoxicity of CuONPs was tested against two human lung cancer cell lines A549 and H1299, as well as apoptotic studies were studied. Furthermore, the photocatalytic activity was done to promote the use of *Phormidium* derived CuONPs as an eco-friendly aspect capable of effectively combating dye degradation.

## Results

### GC–MS analysis of cyanobacterial cell extract

Based on the retention time, the peaks of mass spectra were identified with the help of NIST/Wiley Library. GC–MS profile of *Phormidium sp*. cell extract showed 19 compounds—fatty acid esters (94.5%), carboxylic acid (1.42%), esters (0.84%), alkenes (1.75%), phenolics (0.29%), alkanes (1.40%) and others (Fig. S1; Table S1). Three major peaks of fatty acid esters were observed i.e. Peak 1 with area 21.09% for 9, 12-Octadecadienoic acid, methyl ester, Peak 2 with area 17.06% for 9-Octadecenoic acid, methyl ester and peak 3 with area 12.7% for Hexadecanoic acid, methyl ester.

### Biosynthesis of CuONPs

**Figure 1:**
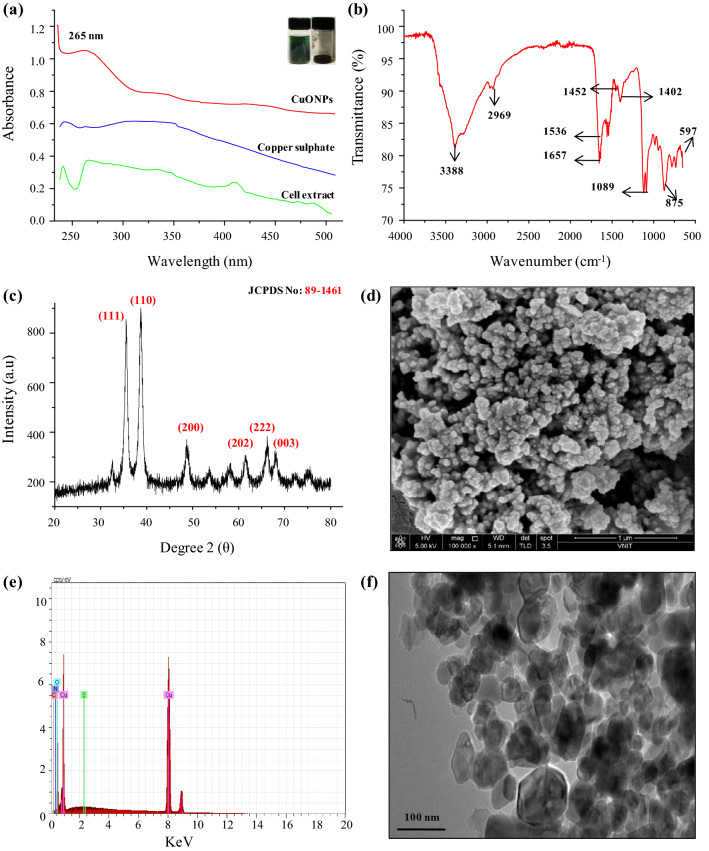
Characterization of *Phormidium sp*. derived CuONPs; (**a**) UV–visible spectrum; (**b**) FTIR spectrum; (**c**) X-ray diffractogram; (**d**) SEM analysis at a direct magnification of × 10 000; (**e**) EDX graph and (**f**) TEM micrograph. The inset in panel (**a**) depicts the green color of cell extract and powder form of CuONPs.

Change in color of the solution from dark green to dark brown indicated the synthesis of CuONPs (Fig. S2). Cyanobacteria derived CuONPs exhibited the maximum absorption peak at 265 nm that confirmed the formation of CuONPs in the solution and no absorption band was determined for copper sulphate and the cell extract at the same region (Fig. 1a).

### Physicochemical characterization of CuONPs

#### FTIR analysis

FTIR analysis of CuONPs and *Phormidium sp*. cell extract was done (Fig. 1b). The broad peak observed at 3388 cm^−1^ in the cell extract corresponds to the –OH stretching of phenolic compounds in the cell extract. The fine peak at 2969 cm^−1^ was attributed to the –CH_2_and C−H stretching mode in alkanes. The intense peak observed around 1657 cm^−1^ can be allocated to the stretching vibrations of proteins (amide I) whereas the band at 1536 cm^−1^is characteristic of amide II. The peak at 1452 cm^−1^ is characteristic to the C-N stretching of aliphatic amines. The peak around 1402 cm^−1^ shows the presence of −COO carboxylic acid present in the cell extract. The sharp peak at 1089 cm^−1^ corresponds to the presence of the stretching vibrations of carboxylic acids and amino groups. The narrow peak observed at 597 cm^−1^ can be attributed to the bending vibrations of CuO, confirming the formation of highly pure CuONPs.

#### XRD analysis

XRD analysis showed the prominent peaks at 35.5°, 38.7°, 48.7°, 53.6°, 58.2° and 61.5° that are attributed to the Miller- Bravais indices of (111), (110), (200), (202), (222), and (003), respectively (Fig. 1c). The observed diffraction peaks of the CuONPs comply well with those of the Joint Committee on Powder Diffraction Standards (JCPDS, card No. 89–1461) pattern of CuO which can be indexed to the hexagonal wurtzite crystal structure of the CuONPs. The detailed information of full-width half maxima (FWHM), Miller indices, d-spacing in nanometer, D- granule size of biogenic CuONPs were indexed in (Table S2). According to the most intense diffraction peak at 2θ = 38.7° (110), the average particle size of CuONPs was found to be 22.5 nm using Debye–Scherrer equation^19^. Moreover, no extra peaks were detected in XRD pattern presenting that CuONPs synthesized by the biogenic method is significantly pure.

#### SEM, TEM and AFM analysis

**Figure 2:**
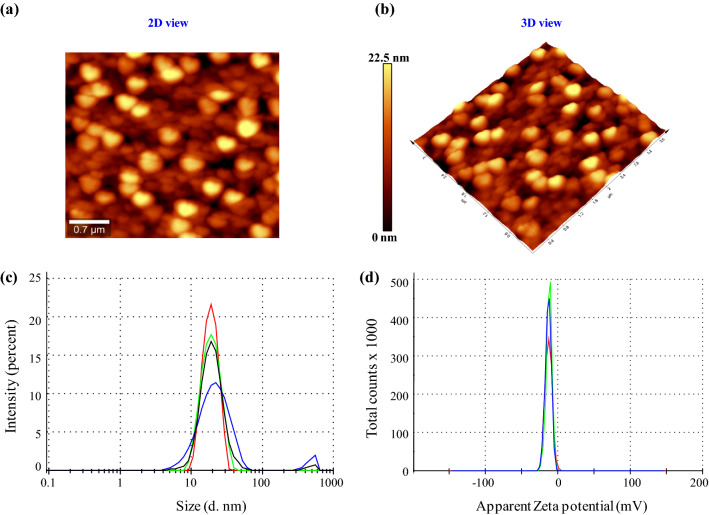
Atomic force microscopy (AFM) results of *Phormidium* derived CuONPs (**a**) Unfiltered AFM image showing topographical 2D image; (**b**) 3D image. Particle size analysis of CuONPs (**c**) Size distribution through Zeta size analyzer; (**d**) Zeta potential distribution.

SEM analysis showed 28.5 ± 2.5 nm sized CuONPs (Fig. 1d). EDX spectrum gave a strong signal at 8 keV with 96% weight of copper (Fig. 1e), which signified the presence of copper. The average size of CuONPs by TEM was found to be 20.7 ± 2.2 nm with spherical or oval shape (Fig. 1f). Tip-corrected AFM analysis showed spherical/oval shaped CuONPs in the range of 0–22.5 nm. The result showed the 2D and 3D view of the sample surface over a 0.7 × 0.7 µm scan and uniform height distribution (Fig. 2a,b).

#### Particle size distribution and zeta potential analysis

DLS analysis suggested the CuONPs size distribution in the range of 68.7 ± 3.4 nm (Fig. 2c). The substances adsorbed on the surface of the nanoparticles (e.g., stabilizers) and the thickness of the electrical double layer (solvation shell), moving along with the particle makes the size bigger in comparison with SEM and TEM microscopic techniques^20^. The polydispersity index (PDI) of CuONPs was 0.220 which pointed out that these particles are moderately dispersed. As per the manual Malvern Zeta analyzer the PDI value less than 0.05 represent highly mono-dispersed distributions of NPs and the PDI value more than 0.7 indicate poly-dispersed distributions of NPs. The calculations used for determination of size and PDI parameters are defined in the ISO standard documents 13,321:1996 E and ISO 22,412:2008 (Manual—Malvern Zeta Size Analyzer)^21^. The zeta potential of CuONPs was found to be − 21.1 mV (Fig. 2d).

### Biological characterization of *Phormidium sp.* derived CuONPs

#### Antioxidant activities of *Phormidium* derived CuONPs

**Figure 3:**
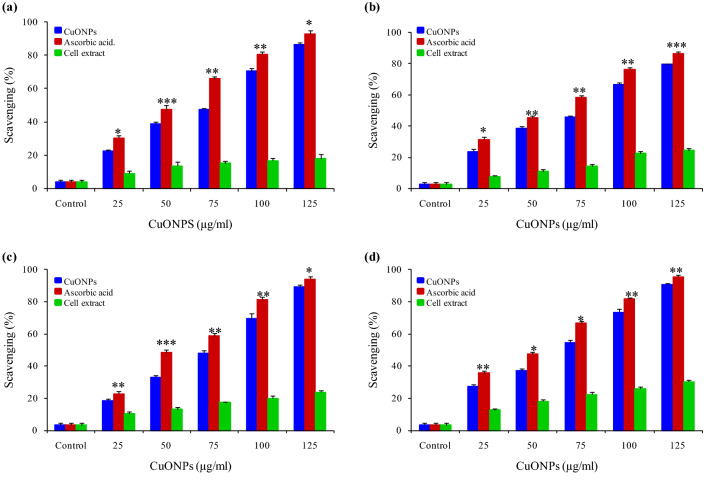
Antioxidant activity of cell free extract, biogenic CuONPs and ascorbic acid by different scavenging assays; (**a**) ABTS; (**b**) DPPH; (**c**) Hydrogen peroxide; (**d**) SOR. Experiments were performed in triplicates; bars represent the mean of values and error bars represent mean ± SD (*P < 0.05).

In the present study the antioxidant activity of aqueous cell free extract, CuONPs and standard ascorbic acid was compared by ABTS, DPPH, SOR and H_2_O_2_ assays and calculated the concentrations at which 50% scavenging (IC_50_) of free radicals was done. Their free radical scavenging activities increased in dose dependent manner (Fig. 3 and Table 1). The order of antioxidant activity (% inhibition of scavenging) was: SOR > H_2_O_2_ > DPPH > ABTS. The IC_50_ value calculated from the percentage inhibition of free radical scavenging revealed that CuONPs showed higher IC_50_ values than standard ascorbic acid but lower than cell free extract indicating their superior antioxidant nature.

**Table 1:** Antioxidant activity of biosynthesized CuONPs.

Sample name	IC_50_ values (µg ml^−1^)			
	ABTS assay	DPPH assay	SOR assay	H_2_O_2_ assay
Aqueous cell extract	201.5 ± 3.33	178.03 ± 1.15	144.7 ± 2.13	170.3 ± 2.41
Biogenic CuONPs	80.1 ± 1.65	77.5 ± 1.60	64.6 ± 1.36	75.8 ± 1.60
Ascorbic acid (Std.)	5.12 ± 2.46	6.68 ± 1.49	5.6 ± 1.20	7 ± 1.60

Average of three independent determinations.

Steady inhibition of ABTS^·+^ free radical (3.9% ± 0.5 to 86.33% ± 0.8) by CuONPs was observed in the concentration ranging from 25 to 125 µg ml^−1^ and corresponding IC_50_ values for CuONPs, extract and ascorbic acid (Std.) were calculated (Fig. 3a and Table 1). DPPH radical scavenging activity at concentration range 25–125 µg ml^−1^ showed scavenging percentage ranging from 23.5% ± 1.08 to 79.6% ± 0.4 which directly depends on hydrogen donating tendency of sample to DPPH radical and corresponding IC_50_ values were calculated (Fig. 3b and Table 1).

#### Antimicrobial activity of CuONPs

**Figure 4:**
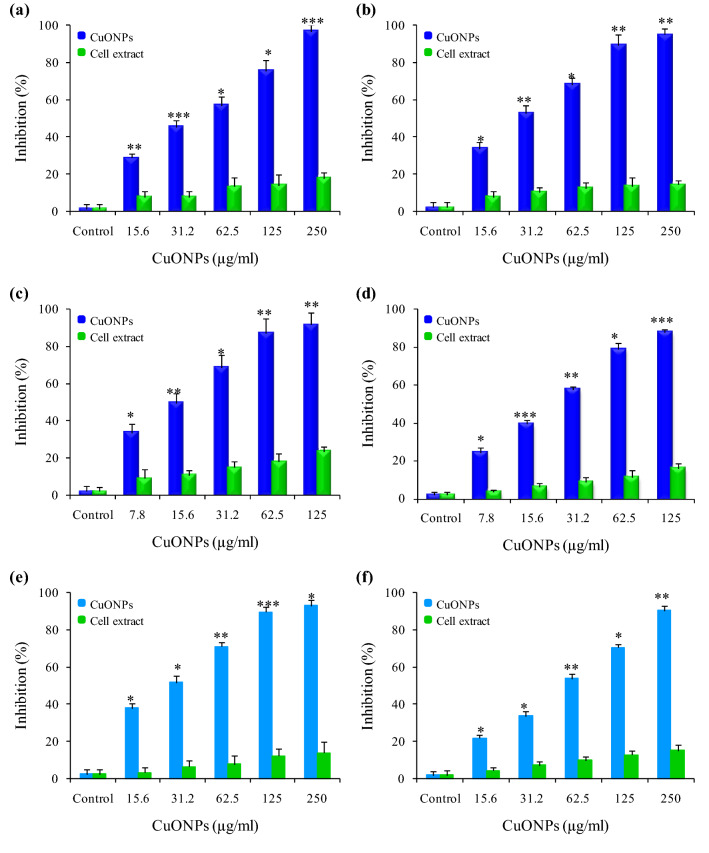
The inhibitory effect of *Phormidium sp*. derived CuONPs and cell extract on; (**a**) *E. coli*; (**b**) *K. pneumonia*; (**c**) *B. cereus*; (**d**) *S. aureus*; (**e**) *C. albicans* and (**f**) *C. glabrata*. Experiments were performed in triplicates; bars represent the mean of values and error bars represent mean ± SD (*P < 0.05).

Biogenic CuONPs exhibited significant dose dependent inhibition for all microbial pathogens (Fig. 4a–d). Higher inhibition was observed against Gram positive (*Bacillus cereus, Staphylococcus aureus*) than gram negative (*Escherichia coli, Klebsiella pneumoniae*) bacteria with lowest MIC value of 62.5 µg ml^−1^ against *B. cereus* (Table 2). Similar results were observed against fungal pathogens as percentage inhibition with maximum inhibition against *C. albicans* having MIC value of 125 µg ml^−1^ (Fig. 4e,f; Table 3).

**Table 2:** Minimum inhibitory concentration (MIC) against pathogenic bacteria alone and in combination with antibiotic (streptomycin) along with interaction index.

MIC (µg ml^−1^)			Interaction index			
Bacteria	CuONPs	Streptomycin	FIC (µg ml^−1^)		FIC index	Interaction mode
			CuONPs + Streptomycin			
*E. coli*	250 ± 1.56	31.2 ± 0.26	15.6 ± 058	3.9 ± 1.51	0.18 ± 0.22	Synergistic
*K. pneumonia*	125 ± 2.25	15.6 ± 1.87	7.8 1 ± 1.19	1.95 ± 1.48	0.07 ± 0.08	Synergistic
*S. aureus*	125 ± 1.21	7.8 ± 1.47	7.81 ± 1.23	0.97 ± 1.77	0.18 ± 0.11	Synergistic
*B. cereus*	62.5 ± 0.51	7.8 ± 1.11	3.9 ± 1.39	0.48 ± 0.37	0.12 ± 0.78	Synergistic

Experiments were performed in triplicates; mean ± SD are shown.

Average of three independent determinations.

**Table 3:** Minimum inhibitory concentration (MIC) against pathogenic fungi alone and in combination with antifungal (fluconazole) along with interaction index.

MIC (µg ml^−1^)			Interaction index			
Fungi	CuONPs	Fluconazole	FIC (µg ml^−1^)		FIC index	Interaction mode
			CuONPs + Fluconzole			
*C. albicans*	125 ± 1.56	31.2 ± 0.26	7.81 ± 0.06	3.9 ± 0.45	0.18 ± 1.12	Synergistic
*C. glabrata*	250 ± 2.25	15.6 ± 1.87	31.2 ± 0.85	3.9 ± 1.04	0.37 ± 0.09	Synergistic

Experiments were performed in triplicates; mean ± SD are shown.

Average of three independent determinations.

**Figure 5:**
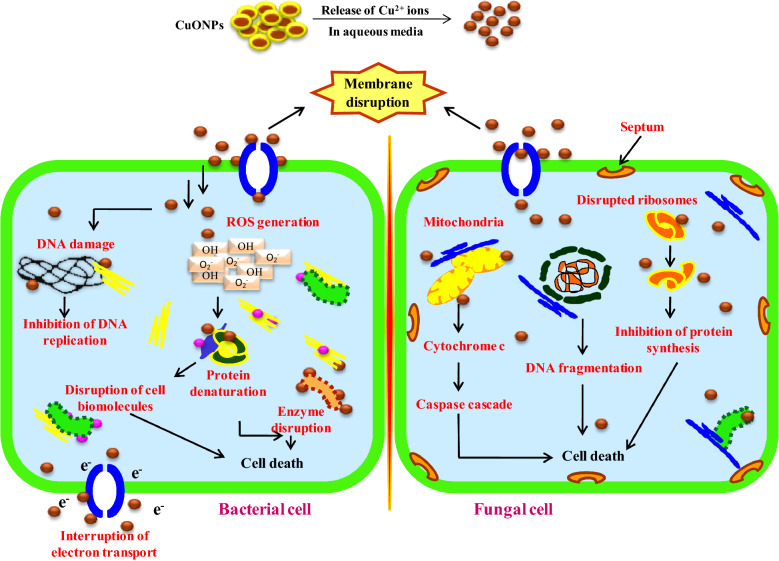
The proposed antimicrobial mechanisms induced by CuONPs for inhibition in bacterial and fungal cells.

Furthermore, synergistic action was determined to describe the interaction of antimicrobial agents, in which the effect produced by the drugs in combination was greater than their individual effects^22^. The interaction indexes (synergistic/antagonistic) for each combination was determined by checkerboard methods. The fractional inhibitory concentration (FIC) indexes against pathogenic bacteria in combination with CuONPs and streptomycin were calculated (Table 2). The degree of synergy was higher for Gram negative species than Gram-positive species. Similar results were observed against *C. albicans* and *C. glabrata* with CuONPs and fluconazole (Table 3). The possible mechanism that lies behind the enhanced antimicrobial action of CuONPs were summarized (Fig. 5).

#### Anti-inflammatory activity of biogenic CuONPs

The maximum inhibition of protein denaturation observed was 86.3% ± 0.33 with 150 µg ml^−1^ CuONPs (Fig. S3). Aspirin (acetylsalicylic acid), a standard inflammatory drug showed maximum inhibition 93.8% ± 1.11 at the concentration of 100 µg ml^−1^. The IC_50_ of aspirin and CuONPs were found to be 34.9 ± 0.87 and 89.9 ± 0.21 µg ml^−1^, respectively.

#### Biocompatibility assay of *Phormidium* derived CuONPs

**Figure 6:**
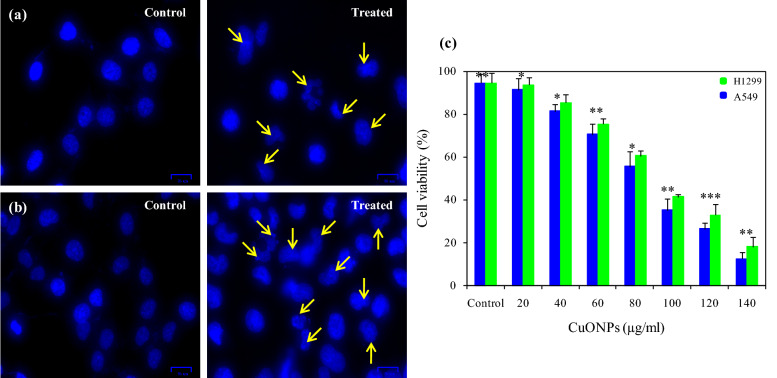
Cytotoxic effect of *Phormidium* derived CuONPs at IC50 concentration against lung cancer cell line (**a**) H1299 (control & treated); (**b**) A549 (control & treated); (**c**) cytotoxic effect of CuONPs against A549 & H1299 lung cancer cell lines at different concentration. Experiments were performed in triplicates; bars represent the mean of values and error bars represent mean ± SD (*P < 0.05).

Biocompatibility of CuONPs against the non-small cell lung cancer cell lines (H1299 and A549) was assessed by MTT assay. CuONPs reduced A549 and H1299 cancer cell line viability in a dose-dependent manner after 24 h exposure (Fig. 6c).

The IC_50_ values calculated for biogenic CuONPs was 100.8 ± 1.24 µg ml^−1^ for H1299 and 88.3 ± 0.74 µg ml^−1^ for A549 cell line. The IC_50_ values for standard drug (doxorubicin) was 0.94 ± 0.55 μg ml^−1^ against H1299 and 1.80 ± 0.05 μg ml^−1^ for A549 cells, respectively. CuONPs treatment induced apoptosis leads to the conformational changes visualized by DAPI staining as nuclei blebbing, condensation and cracking constitutes the characteristic features of apoptosis (Fig. 6a,b). Cells without treatment (control) had shown intact nuclei with smooth edges having uniform shape and size in both the cell lines.

#### Dye degradation by CuONPs

**Figure 7:**
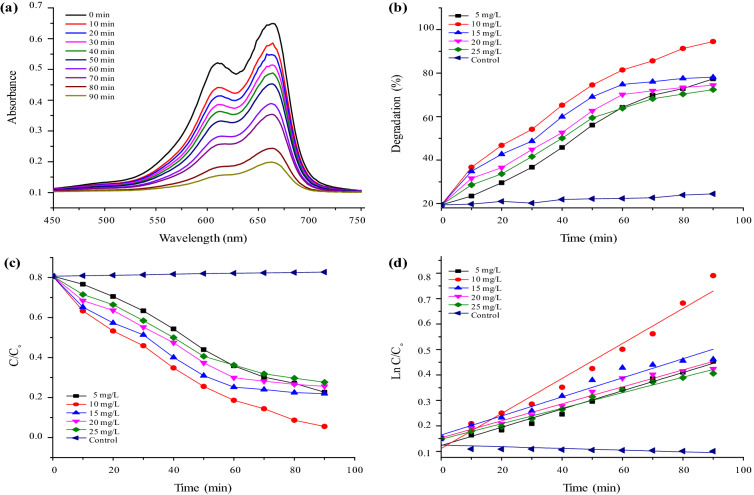
Photodegradation of methylene blue (MB) dye, (**a**) Effect of solar irradiation using CuONPs on photocatalytic degradation of MB dye; (**b**) Percent photocatalytic degradation of MB dye as effect of time with varying photocatalyst (CuONPs) load; (**c**) Photodegradation of dye versus irradiation time at varying photocatalyst (CuONPs) measured by optical absorption; (**d**) Plot of ln(C_0_/C) versus irradiation time for dye degradation kinetics.

To investigate the photocatalytic activity of CuONPs the organic dye MB was subjected to photodegradation using biogenic CuONPs (5, 10, 15, 20 and 25 mg/L). The control kept in dark, showed no visual color change. But the reaction mixtures supplemented with CuONPs in sunlight exhibited change in color from dark blue to pale blue. UV–Vis scanning of the reaction suspension for MB at 665 nm showed that the peak height decreased with increase in time that suggested increased dye degradation with time (Fig. 7a). Maximum degradation of MB dye (94%) dye was observed after 90 min (Fig. 7b).

For the identification of CuONPs induced free radical during the photodegradation of dye, elimination studies were performed by the addition of ammonium oxalate (AO) as h^+^ scavenger, *p*-benzoquinone (*p*-BQ) as O_2_^−^ scavenger and tert-butanol (*t*-BuOH) as OH scavenger in the reaction suspensions. The addition of AO exhibited the minimal change in the photocatalytic degradation of dyes indicating that h^+^ radicals played very little role in degradation (Fig. S4). The addition of p-BQ (·O_2_^−^ scavenger) exhibited higher reduction than h^+^ radicals indicates their relatively higher role in dye degradation. However, the degradation rate of MB dye reduced maximally upto 65% with the addition of t-BuOH (OH^−^ scavenger) and suggested that hydroxyl radicals were mainly involved in dye degradation. The decrease in the removal rate in the presence of scavengers presents the following trend:* t*-BuOH > *p*-BQ > AO. To quantitatively study the photocatalytic activity of CuONPs, we performed the plots of ln(C/C_0_) versus irradiation time, assuming that the degradation reaction of dye by CuONPs under visible light irradiation followed the pseudo-first-order kinetics. It can be modeled as:

$$\mathrm{ln}\frac{{\mathrm{C}}_{0}}{{\mathrm{C}}_{\mathrm{t}}}={K}_{abs}t$$

where K_abs_ is the photodegradation rate constant (min^−1^), C_0_ is the initial concentration of MB and dye and C_t_ is the concentration of dye at time *t*^23^. Using MB dye initial concentration of 25 mg/L and varying photocatalyst load, plots of best-fit lines of ln(C_0_/C_t_) versus time were plotted (Fig. 7c,d). The linear nature of our data confirmed that the photodegradation of MB dye using photocatalysts fits well with the kinetic model. It is worth noting that dye concentration in these experiment remains in the optical regime where the Beer–Lambert law holds. All correlation coefficient (R^2^) values were higher than 0.95 with an average value of 0.96 for photocatalytic degradation of MB dye. Our results confirmed the applicability of the pseudo first-order reaction kinetic model for photocatalytic degradation of MB dye. Assuming the reaction kinetics, highest rate constant (*k*) determined from the slope of ln(C_0_/Ct) versus time linear plots, corresponding to optimum catalyst load (CuONPs) of 10 mg/L, was 0.026 min^−1^ for MB dye.

## Discussion

Biogenic CuONPs synthesis got worldwide attention due to their wide applications in biomedicine. During the present investigation, CuONPs had been synthesized using the crude cyanobacterial (*Phormidium*) cell free aqueous extract. Extra-cellular biosynthesis of CuONPs is much economical than intra-cellular synthesis over the use of chemicals, energy and time required for cell disruption and purification. GC–MS analysis of *Phormidium* cell extract showed the presence of several active principle compounds that may help in the synthesis of CuONPs. These compounds were also previously reported in the GC–MS profile of *Nostoc sp. EA03*^24^, and *Synechocystis sp.* PCC 7338^25^, and suggested their reducing nature. Fatty acid esters, viz., Octadecanoic acid, methyl ester and 13-Docosenoic acid, methyl ester were found in lesser quantity (1.74% and 0.64% respectively). The derivatives of these fatty acid esters with reducing potential have been also found in *Allium saralicum*^26^ and *Citrus wax*^27^. Esters like heptadecanoic acid- methyl ester and Octadecenoic acid (Z)—methyl ester were also found in cyanobacterial extract. Heptadecanoic acid, methyl ester from *Mentha spicata*^28^ and derivative of octadecenoic acid (Z)-, methyl ester from *Thesium humile*^29^ were also reported for their good reducing potential. Apart from the esters, few carboxylic acids i.e. Methacrylic acid (0.13%) and 1, 2-benzenedicarboxylic acid (0.22%) were also identified in extract. Previously, the reducing nature of these two carboxylic acids was reported in *Calothrix brevisima*^30^ and *Punica* species^31^. Compounds of other classes like alkane (Nonadecane), alkene (Neophytadiene, 1, 3, 12-Nonadecatriene), cycloalkene derivatives (cyclododecyne, tricycle [20.8.0.0(7,16)] triacontan and 1,1':3',1"-tercyclopentane, 2'-dodecyl), derivative of acetanilide (acetamide, N-(3,5-diaminophenyl) and phenol (Phytol) have been also obtained in the present study using *Phormidium sp.* cell extract. During previous studies some related compounds were also reported with good reducing ability like, Neophytadiene from *Plectranthus amboinicus*^32^, 5-(1,5-dimethyl-4-hexenyl from *Zingiber officinal*^33^, Phenol, 3,5-bis(1,1-dimethylethyl from *Calothrix brevisimma*^30^. The phyto-chemicals present in cyanobacterial extract such as chlorophyll, carotenoids, phycobilliproteins, flavonoids, phenols and proteins act as both reducing as well as stabilizing (capping) agents for NPs synthesis^34^. The flavonoids have the ability to donate the hydrogen or electron, and the phenolics exhibit a chelating effect on the metal ions, which is responsible for the reduction of Cu^2+^ from CuSO_4_.5H_2_O to copper oxide nanoparticles^35^. Proteins present in the extract bind to nanoparticles through free amine groups or cysteine residues or negatively charged carboxylate groups of enzymes that help in synthesis as well as stabilization of nanoparticles. Exact chemical nature of reducing and capping agent is not yet explored for CuONPs. But with regard to CdS nanoparticles derived from *Phormidium tenue NTDM05*, fluorescent phycobilin protein are reported to prevent agglomeration of nanoparticles^36^. CuONPs synthesized from cyanobacteria exhibited different λ max, e.g. at 259 nm in *Spirulina platensis*^37^, 600 nm in *Phormidium sp*.^38^, 220 nm in *Cylindrospermum stagnale*^39^ and 580 nm in *Spirulina platensis*^40^. Furthermore, CuONPs from other organisms have showed slightly different λ max, e.g. at 282 nm in *Punica granatum*^41^, 250 nm in *Achillea millefolium*^3^, 260 nm in *Aloe vera*^42^, 265 nm in *Aloe barbadensis Miller*^43^, 250 nm in *Caesalpinia bonducella*^44^, 245 nm in *Momordica charantia*^5^ and 277 nm in *Citrofortunella microcarpa*^45^.

Biogenic CuONPs were further purified for the removal of unwanted biomolecules by centrifugation after 3 h. Then physical characterization was done by XRD, SEM, EDX, TEM, AFM and DLS analysis. In FTIR analysis peaks obtained in *Phormidium sp*. derived CuONPs showed functional groups that may have served as the reducing and capping agents during its synthesis (Fig. 1b). Similar observations were also reported by other researchers e.g. peak at 574 cm^−1^ in *Millettia pinnata* flower extract^4^, at 540 cm^−1^ in aqueous black bean extract^46^, peak at 576 cm^−1^ in brown alga *Cystoseira trinodis*^11^, 590 cm^−1^ in *Momordica charantia* aqueous extract^5^ and 529 cm^−1^ in *Aloe vera* leaves extract^42^. The peak observed at 875 cm^−1^ may be attributed to the aromatic C-H bending. In *Citrofortunella microcarpa* leaves extract identical peak at 880 cm^−1^ was reported^45^. This indicates that the biological molecule present in the cell extract has a dual function of stabilization and formation of CuONPs. XRD analysis of CuONPs from *Phormidium sp.* cell extract showed well-defined peaks at 2θ value that indicated their crystalline nature (Fig. 1c). When the crystalline size decreases from bulk to nanoscale dimensions, the XRD peaks broaden^19^. XRD result showed that CuONPs formed are crystalline in nature with average size 22.5 nm close to particle size measured by TEM. Several biologically synthesized CuONPs also showed the crystalline nature viz., from cyanobacteria *Phormidium*^47^, *Spirulina platensis*^37^, brown alga *Cystoseira trinodis*^11^ and *Bifurcaria bifurcate*^48^. Higher plants also exhibited crystalline nature of biosynthesized CuONPs e.g. *Aloe barbadensis Miller* leaves extract^43^, *Caesalpinia bonducella* seed extract^44^ and *Achillea millefolium* leaf extracts^3^. Scanning electron microscopy with energy dispersive X-ray (SEM–EDX) was done to confirm the presence of elemental copper in biologically synthesized CuONPs. The presence of copper (96%) and oxygen in EDX analysis confirmed the purity of synthesized CuONPs (Fig. 1d,e).

TEM and AFM analysis were also done for size and surface topology (Figs. 1f and 2b). CuONPs of different shapes and sizes had been reported from cyanobacteria e.g. *Phormidium*, *Spirulina platensis* and *Bifurcaria bifurcate* synthesized quasi-spherical shaped CuONPs in the range of 40–45 nm^37,47,48^. Other studies on higher plant mediated synthesis have reported lesser purity e.g. CuONPs synthesized from *Aloe vera* extract exhibited 54% of elemental copper having size of 30 nm with some larger particles (100 nm)^42^. CuONPs synthesized from *Momordica charantia* showed an average size of 61.4 nm with 54.5% of copper^5^. Additionally, large sized spherical CuONPs have been reported from *Citrofortunella microcarpa* (68–75 nm)^45^ and from actinomycetes (61.7 nm)^49^. Dynamic Light Scattering (DLS) was used to determine hydrodynamic sizes, polydispersities and aggregation effects of colloidal sample based on the Brownian movement^20^. It is the particle diffusion behavior within any fluid, measured by the fluctuations in light intensity that passes through a colloidal solution as a function of time^50^. The CuONPs size distribution in the range of 68.7 ± 3.4 nm (Fig. 2c). The obtained PDI value of 0.2 for CuONPs suggested their intermediate or moderate nature. As per the manual Malvern Zeta analyzer the PDI value less than 0.05 represent highly mono-dispersed distributions of NPs and the PDI value more than 0.7 indicate poly-dispersed distributions of NPs^21^. Further, zeta potential measurement was done to check the stability of synthesized CuONPs (Fig. 2d). It is considered as a measure of charges on the surface of nanoparticles. Large positive (> + 30 mV) or negative (≥ 30 mV) charges tends to repel each other whereas low zeta potential value causes the aggregation of NPs due to absence of repulsive force and provide stability to the nanoparticles^17^. Qamar et al.^5^ synthesized CuONPs from *Momordica charantia* leaf extract with zeta potential of − 7.23 mV only. The more negative value of zeta potential of the *Phormidium* derived CuONPs suggested their higher stability.

Cyanobacteria and many medicinal plants possess free radical scavenging moieties such as phenolic compounds, terpenoids, flavinoids, vitamins and other endogenous metabolites, that are rich in antioxidant activity^51^. *Phormidium* derived CuONPs exhibited higher percentage of free radicals scavenging. Cellular respiration leads to the production of reactive oxygen species (ROS) and several other free radicals having unpaired valence shell electrons. These free radicals play vital role in cell signaling but in excess leads to oxidative damage to the cell by reacting with the cellular components that cause cancer, aging, cataract, cardiovascular diseases and dysfunction of organs. Thus, antioxidants play a crucial role to down regulate and eliminate free radicals before they damage the cell^52^. The present study showed the antioxidant activity of CuONPs by ABTS, DPPH, SOR and H_2_O_2_ assays. ABTS^·+^ is a pre generated free radical and the interaction between antioxidant and ABTS^·+^ causes bleaching of ABTS^·+^^17^. CuONPs exhibited percentage inhibition from 3.9 to 86.33% and corresponding IC_50_ values for CuONPs, extract and ascorbic acid (Std.) were calculated (Fig. 3a and Table 1). Kumar and Shanmugam^53^ also reported 88% ± 1.12 inhibition of ABTS^·+^ free radicals with 500 µg ml^−1^ of CuONPs synthesized by using *Magnolia champaca* floral extract.

In DPPH radical scavenging, CuONPs exhibited percentage inhibition ranging from 23.5 to 79.6% and corresponding IC_50_ values were calculated (Fig. 3b and Table 1). In previous reports, *Cystoseira trinodis* (brown algae), *Solanum nigrum* and *Mussaenda frondosa* derived CuONPs exhibited 50% scavenging inhibition with an IC_50_ value of 543 µg ml^−1^, 131.5 µg ml^−1^ and 1570 µg ml^−1^, respectively^8,11,54^.

A significant dose dependent inhibition was observed with biogenic CuONPs against bacterial strains (Fig. 4a–d). Karuppannan et al.^55^ synthesized CuONPs from *Cardiospermum halicacabum* extract exhibited the antibacterial action with MIC value of 1 mg ml^−1^ for *S. aureus* and *E.coli*. Manasa et al.^54^ synthesized CuONPs from *Mussaenda frondosa L* that exhibited antibacterial potential with MIC value of 305 µg ml^−1^ against *E. coli.* Furthermore, CuONPs synthesized from brown algal extract of *Bifurcaria bifurcate*^48^ and cyanobacteria *Spirulina platensis* extract^37^ showed antibacterial potential against *S. aureus*, *B. cereus, E. coli* and *K. pneumonia* by disc diffusion. CuONPs synthesized from actinomycetes^49^, *Achillea millefolium* leaf extracts^3^ and *Momordica charantia*^5^ exhibited the antibacterial activity with maximum zones of inhibition. However mechanically synthesized CuONPs also showed the antibacterial activity but with higher MIC value of 2.5 mg ml^−1^ for *S. aureus* and 3.75 mg ml^−1^ for *E.coli*^56^. CuONPs synthesized using *Psidium guajava* leaf extract exhibited enhanced antibacterial activity against gram negative (*E. coli, P. aeruginosa*) and gram positive (*S. pneumoniae, S. epidermidis*) bacterial strains using agar well diffusion, and CuONPs interaction with bacterial membrane was shown by confocal laser scanning microscopic (CLSM)^57^. CuONPs synthesized using *Abutilon indicum* leaf extract showed antibacterial activity against *E. coli, S. typhi, B. subtilis* and *S. aureus* bacteria by using agar well diffusion method^58^. Limited studies were known in the literature on the antifungal activity of CuONPs with reference to *Candida* species. CuONPs synthesized from *Achillea millefolium* leaf extracts^3^, *Brassica oleracea var.* extract^59^ and chemically synthesized CuONPs^60^ exhibited the potent antifungal activity against *C. albicans* and *C. glabrata*. However, commercially synthesized CuONPs showed the antifungal activity with MIC value of 1 mg ml^−1^ for *C. albicans* and *C. glabrata*^61^. Our results highlight the presence of synergistic interactions between CuONPs and antibiotic/fungicides combinations (Tables 2 and 3). The possible mechanism behind the enhanced synergistic antimicrobial action of CuONPs may be due to the involvement of active functional groups such as hydroxyl and amino groups present in the antibiotics/fungicides that can be chelated by CuONPs^22,62^. Since CuONPs has potential antimicrobial properties with synergistic potential they may be used in food preservation and packaging. The antimicrobial mechanism of CuONPs may be due to the generation of reactive oxygen species (ROS) generation that disrupts the cell membrane causing direct cellular toxicity^63^. The possible mechanisms involved by the CuONPs induced antimicrobial activity can be summarized as (Fig. 5). The carboxylic and amines group present in cell membrane facilitates the penetration of CuONPs by the intracellular dissolution of soluble copper ions and accumulation of superoxides or hydroxyl radicals that leads to oxidative stress. CuONPs generates potent cytotoxicity by DNA damage, mitochondrial degradation, ribosomes disruption and dysfunction of different proteins channels present in cell membrane that leads to cell death^64,65^.

Inflammation is a complex process, associated with pain and involves increased protein denaturation, vascular permeability and membrane alteration. During denaturation, proteins lose their secondary and tertiary structure due to stress or heat that causes inflammation. Limited reports are available on the anti inflammatory activity of CuONPs. Maximum protein denaturation observed was 86.3% with 150 µg ml^−1^ CuONPs (Fig. S3) and the IC_50_ of aspirin and CuONPs were found to be 34.9 ± 0.87 and 89.9 ± 0.21 µg ml^−1^, respectively. Thiruvengadam et al.^4^ observed 80% of albumin denaturation by the CuONPs synthesized using *Millettia pinnata* flower extract. Biosynthesized CuONPs using *Triumfetta rotundifolia* exhibited 57.4% of human RBCs membrane stabilization at 1 mg ml^−1^ and 58% at 100 mg ml^−1^ using *Mussaenda frondosa L*. extract^54,66^. CuONPs synthesized using *Bacopa monnieri* leaf extract exhibited the in-vivo anti-inflammatory activity by 74% inhibition of edema in comparison to diclofinac sodium as standard by 24% after 48 h^67^. CuONPs synthesized using *Cissus quadrangularis* leaf extract showed the enhanced anti-inflammatory activity in membrane stabilization and anti-proteinase activity in comparison to standard drug^68^. Chemically synthesized CuONPs were also tested for their anti-inflammatory activity both in-vitro and in-vitro on albino mice. Results showed that BSA denaturation was highly inhibited by CuONPs in the in-vitro anti-inflammatory activity and in-vivo activity revealed that low doses (5 mg/kg) of CuONPs were more potent in inhibiting inflammation than high doses (10 and 20 mg/kg) of free drugs^69^.

Cytotoxicity of CuONPs against the non-small cell lung cancer cell lines (H1299 and A549) was assessed by MTT assay. Appreciable anticancer activity was observed against A549 cells with lower IC_50_ value than H1299 cells (Fig. 6c). DAPI staining was done to determine the apoptosis caused by CuONPs on cell lines that showed the nuclei blebbing and condensation in treated cells (Fig. 6a,b). Manasa et al.^54^ showed an IC_50_ value of 218.18 µg ml^−1^ from the biosynthesized CuONPs using *Mussaenda frondosa L*. extracts against A549 cancer cells. The CuONPs synthesized from *Ficus religiosa* extract evaluated against A549 cell line by MTT assay showed an IC_50_ value of 200 μg ml^−1^^70^. Green synthesized CuONPs from *Ficus religiosa* leaf extract inhibited histone deacetylases (HDAC) level and exhibited apoptosis mediated anticancer activity against A549 lung cancer cell line with an IC_50_ value of 200 μg ml^−1^^71^. CuONPs synthesized using *Abutilon indicum* leaf extract exhibited the cytotoxicity against human lung A549 and breast MDA-MB-231cancer cell lines through MTT assay^58^. CuONPs-mediated anticancer activity involves oxidative stress, ROS accumulation, chromosomal aberration, genetic material fragmentation, caspase production, and enhancement of intrinsic and extrinsic apoptotic pathways^64^. CuONPs caused DNA damage, halted cell cycle, and inhibited cell proliferation in cervical cancer HeLa cells^72^. CuONPs synthesized using marine entophytic actinomycetes showed the cytotoxicity against A549 lung cancer cells at concentration of 500 µg ml^−1^ with 54% inhibition and an irregular morphology of cancer cells^73^. Furthermore, Singh et al.^74^ and Elsayed et al.^75^ observed green synthesized CuONPs as an efficient antiproliferative activity in the treatment of targeted breast cancer. The lower IC_50_ values of *Phormidium* derived CuONPs against cancer cell lines (significant at *P* ≤ 0.05) clearly put forward the fact that biosynthesized CuONPs have potent cytotoxicity.

The photocatalytic activity of CuONPs was done using MB dye at different concentrations (5, 10, 15, 20 and 25 mg/L) showed change in color from dark blue to pale blue. UV–Vis scanning exhibited the reaction peak for MB at 665 nm (Fig. 7a). The percent degradation under visible light irradiation observed was 94% after 90 min (Fig. 7b) and was calculated by the corresponding percent adsorption value using the equation given by Kwon et al.^76^. Sinha and Ahmaruzzaman.^77^ reported that CuONPs (10 mg/L) synthesized from aqueous extract of *L. rohita* scales degraded 96% of MB dye in 135 min and CuONPs (50 mg/L) synthesized from brown algae *Cystoseira trinodis* degraded 89% of MB dye in 210 min^11^. Biogenic CuONPs (20 mg/L) synthesized from plant extracts of *Mussaenda frondosa* leaf extract exhibited 97% of MB degradation in 140 min^54^ and CuONPs (10 mg/L) from cell extract of *Cardiospermum halicacabum* exhibited 93% degradation in 210 min^55^. CuONPs (0.2 g/L) synthesized from plant extract of *Achillea millefolium*^3^, degraded 96% of MB dye in 120 min. It is necessary to determine which reactive species plays greater role in the photocatalytic degradation process. Biosynthesized CuONPs (20 mg) using *Psidium guajava* leaf extract showed 89% degradation of MB dye in 150 min^57^.

CuONPs induced free radical during the photodegradation of dye, showed in the presence of t-BuOH (OH^-^ scavenger) the degradation rate of MB dye reduced maximally upto 65% (Fig. S4). Sharma and Dutta^78^ also reported that hydroxyl radicals were the most important reactive oxygen species in the dye degradation by specific ROS scavenger test using isopropyl alcohol as scavengers for ·OH radicals. After establishing photocatalytic role of CuONPs by measuring percentage degradation of MB dye, attention was paid for kinetic study. The plots of best-fit lines of ln(C_0_/C_t_) versus time were plotted using MB dye initial concentration (25 mg/L) and varying photocatalyst load, (Fig. 7c,d) that exhibited the linear nature of our data and confirmed the photodegradation of MB dye fits well with the pseudo first-order reaction kinetic model. The higher photocatalytic degradation rate of biosynthesized CuONPs may be due to its smaller particle size (20.7 nm, TEM analysis). Moreover, this can be attributed to the higher density of electron-deficient sites generated during synthesis that can trap photogenerated electrons and reduce recombinations, thereby improving the photocatalytic activity^77^. The probable mechanism for the photocatalytic activity of CuONPs based on the interaction with light source i.e. associated with photon absorption and connectivity among the surface reactivity and, surface radical formation among (H_2_O, O_2_)^3^. The photocatalytic mechanism was initiated when the photons were absorbed by CuONPs, get photo excited and undergoes plasmonic decay. According to Ghareib et al.^79^ complete reaction occurred during photocatalytic degradation of MB dye by CuONPs can be summarized as below:

1 $${\text{Cu}} + {\text{h}}v \to {\text{h}}^{ + } \left( {{\text{Cu}}} \right) + {\text{e}}^{ - } \left( {{\text{Cu}}} \right)$$

2 $${\text{H}}_{{2}} {\text{O}}\left( {{\text{ads}}} \right) + {\text{ h}}^{ + } \to {\text{OH}} + {\text{ H}}^{ + } ({\text{ads}})$$

3 $${\text{O}}_{{2}} + {\text{e}}^{ - } \to {\text{O}}_{{2}}^{ - } \left( {{\text{ads}}} \right)$$

4 $${\text{O}}_{{2}}^{ - } \left( {{\text{ads}}} \right) + {\text{ H}}^{ + } \leftrightarrow {\text{HOO}}^{.} \left( {{\text{ads}}} \right)$$

5 $${\text{2HOO}}^{.} \left( {{\text{ads}}} \right) \to {\text{H}}_{{2}} {\text{O}}_{{2}} \left( {{\text{ads}}} \right) + {\text{ O}}_{{2}}$$

6 $${\text{H}}_{{2}} {\text{O}}_{{2}} \left( {{\text{ads}}} \right) \to {\text{2OH}}^{.} \left( {{\text{ads}}} \right)$$

7 $${\text{Dye}} + {\text{OH}}^{.} \to {\text{CO}}_{{2}} + {\text{ H}}_{{2}} {\text{O }}\left( {\text{dye intermediates}} \right)$$

8 $${\text{Dye}} + {\text{h}}^{ + } \to {\text{Oxidation products}}$$

9 $${\text{Dye}} + {\text{ e}}^{ - } \to {\text{Reduction product}}$$

The electron or holes generated due to plasmonic decay react with O_2_ and, H_2_O molecules to generate active species; anionic super oxide radical (O_2_^−^.) and hydroxyl radical (OH.), respectively. Furthermore, the hydro peroxyl radical (HO_2_) obtained by the protonation of superoxide ion (O_2_^−^) radical converts to H_2_O_2_, that inevitably dissociates into highly reactive hydroxyl radicals (OH). Here, in this process CuONPs play an important role as an electron carrier. The greater degradation efficiency of CuONPs, may be attributed to the reducing agents presents in the algal extract that promotes the formation of oxygen species (e.g. superoxide and hydroxyl radical) upon irradiation ^11^. Such an efficient photodegradation provide a convenient path for the treatment of dyes under visible light irradiation.

## Conclusion and future prospects

In summary the present study emphasizes a cost effective, environmentally benign and bio-inspired protocol for the synthesis of CuONPs followed by its biomedical ad photocatalytic applications. The phytochemicals present in cyanobacteria *Phormidium* cell extract act as a bio-reductant of Cu^2+^ ions as well as a stabilizing agent for biosynthesis of CuONPs. Dark brown coloured, small (20.7 nm), spherical shaped CuONPs with high purity (96%) were successfully synthesized. Towards its biomedical applications, biogenic CuONPs proved its excellent efficiency in the free radical scavenging and exhibited the pronounced toxicity against pathogenic microbial strains with high degree of synergistic interactions. Results of cytotoxicity suggested the therapeutic use of CuONPs in the treatment of inflammatory diseases and human lung cancer. In addition CuONPs exhibited its convenient utilization for the efficient photocatalytic degradation of the carcinogenic methylene blue dye that poses serious threat to human health. However, for the advanced CuONPs biomedical applications, more extensive research is still required to reduce its toxicity while improving its biological efficiency. The applications of CuONPs in the field of sensors, food packaging and antimicrobial action will give its future applications by taking in consideration the safety aspects as a serious concern. The findings of the present study may expand the prospects for the biogenic synthesis of CuONPs and their use in a variety of biomedical and biotechnological applications.

## Materials and methods

### Chemicals

Analytical grade chemicals used were purchased from Sigma-Aldrich (India) or Merck (India). All culture media were purchased from Hi Media (India). All the buffers and reagents used were prepared in double-distilled water (DDW). Glassware were thoroughly washed and air-dried before use.

### Test organism and growth conditions

Cyanobacterial strain *Phormidium sp.* (NCCU-104) was procured from IARI, New Delhi, India. Axenic culture was routinely grown in BG-11 liquid medium in culture room at 30 ± 2 °C and illuminated with cool white Sylvania 40W T12 fluorescent lamps having a lux intensity of 2000 ± 200 for 12 h light/dark cycles^80^. Cyanobacteria were harvested in the mid-exponential growth phase for extract preparation. For antibacterial study, *Klebsiella pneumoniae* (KJ 938546), *Staphylococcus aureus* (MCC 2708), *Bacillus cereus* (MCC 2243), and *Escherichia coli* (MCC 2052) were received from the microbial culture collection of the National Centre for Cell Science (NCCS), Pune, India. Clinical isolates of *Candida albicans* (MCC 1151) and *Candida glabrata* (MCC 1432) were obtained from National Fungal Culture Collection of India (NFCCI), Pune, India. For anticancer study A549 and H1299 (Human NSCLC cell lines) were obtained from the National Centre for Cell Science (NCCS) Pune, India and maintained in DMEM (Cat # 10569010, Gibco, Waltham, MA, USA) and incubated at 37 °C and an atmosphere of 5% CO_2_.

### Preparation of cell extract and its characterization

Thoroughly washed biomass pellet were suspended in 100 ml of sterile water and sonicated in a Branson Sonifier 450 (Branson Ultrasonics Corp., USA) for 3–5 min at maximum output and duty cycles to ensure complete breakage of the cells. If the extract showed the presence of filaments/cells, steps of sonication was repeated. The resulting extracts were centrifuged at 10,000 g for 30 min, filtered through Whatman No. 1 filter paper and stored in refrigerator at 4 °C for further use as cell extract^18^. The chemical profile of cyanobacterial extract was analysed using GC–MS to find out the probable compounds that have reducing potential and aided the synthesis of nanoparticles. Samples for GC–MS analysis were prepared by dissolving the dried extract in methanol. The GC–MS analysis was done by Shimadzu GC–MS QP 2010 Plus equipment in electron ionization (EI) mode fitted with a RTX-5 capillary column (60 m × 0.25 mm × 0.25 μm). Helium was used as carrier gas with 0.7 ml min^–1^of flow rate. The temperature of the injector was fixed at 260 °C. The initial and final temperature of the column was 80 °C and to 280 °C at the rate of 10 °C min^–1^ and 15 °C min^–1^ respectively. A 3.5 min solvent delay was used. Mass spectra were recorded under scan mode in the range of 40–650 m/z. Compounds were identified by comparing with NIST11/ WILEY library^81^.

### Biosynthesis of copper oxide nanoparticles

For the extracellular synthesis of CuONPs, in 10 ml of algal extract, added 90 ml of copper sulphate solution (0.1 M). Together with this, algal extract (without copper sulphate) and copper sulphate solution alone were taken separately in two flasks to serve as positive and negative controls. The reaction mixture was kept under vigorous stirring at 90 °C, with gradual addition of 5 ml of 0.1 M (NaOH) the deep blue solution gradually turned to brick red coloration which changed to dark after vigorous stirring for 3 h. The black solid product obtained was centrifuged thrice at 15,000 rpm for 20 min after thoroughly washed with distilled water and 90% ethanol successively to remove any impurities. The resulting solid precipitate was oven-dried, crushed into powder, and stored in airtight container for further analysis^48,49^. Biosynthesized CuONPs were analyzed by UV–Vis spectrophotometer in 200–500 nm range (Labtronics LT 2900).

### Physiochemical characterization of *Phormidium* derived CuONPs

Biosynthesized CuONPs were characterized for their physicochemical properties with the help of different physical techniques like UV–Vis Spectroscopy, Fourier Transform Infrared Spectroscopy (FTIR), X-ray diffraction (XRD), and Dynamic light scattering (DLS), Scanning Electron Microscopy (SEM) with EDX, Transmission Electron Microscopy (TEM), Atomic Force Microscopy (AFM) and Zeta potential analysis. Biosynthesized CuONPs were analyzed using a Double-beam UV–Vis spectrophotometer (Labtronics LT 2900). The CuONPs suspension was centrifuged at 10,000 rpm/15 min and air dried sample analysis was recorded on the FT-IR spectrophotometer (Perkin Elmer, USA) in the range 500–4000 cm^−1^ at a resolution of 4 cm^−1^^82^. XRD (Rigaku X-ray diffractometer, Ultima IV, Japan) of CuONPs was recorded at 2θ in the range from 20° to 80° at 40 kV/30 mA with CuKa radiation (k ¼ 1.54056°A), and the crystalline domain diameter of CuONPs was calculated from the XRD peaks by the Debye–Scherrer equation:

$$D=\frac{0.94\lambda }{\beta cos\theta }$$

where, D is the crystalline domain diameter of ZnONPs, “λ” is the wavelength of the X-ray source used (0.1541), and “β” is the full width at half maximum (FWHM) of the diffraction peak and “θ” is the Bragg’s angle. Dynamic Light Scattering (DLS) (Malvern, UK) was used to determine hydrodynamic size and polydispersity of nanoparticles. Size distributions of synthesized CuONPs were measured using a Nano Zetasizer system (Zeta sizer, Malvern, UK). Before measuring the sample were passed through a 0.2 μm polyvinylidene fluoride (PVDF) membrane with the parameters used such as measurement temperature (25 °C), medium viscosity, and material refractive index (1.59)^18^. The size of CuONPs was determined by SEM (Carl Zeiss, USA) equipped with EDS, and TEM. For SEM analysis (Sigma version 5.05 Zeiss, USA), thin films of the CuONPs were prepared by dropping an exceedingly small amount of the sample on the silicon wafer at an accelerating voltage of 20 kV with time ranging from 60 to 100 s^82,83^. The scientific instrument Sirius SD model (the EDX spectrometer-equipped SEM) was used to determine the elemental analysis of the CuONPs. TEM analysis was done on (Philips EM-410LS, JEOL, Japan) using 10 μl of the CuONPs solution kept on the carbon-coated copper grids, making a thin film on the grid and kept in a grid box, sequentially^82^. Selected portion of the images were observed using Image J and evaluated to obtain the size distribution histogram. The surface topology of synthesized ZnO NPs was obtained by AFM analysis. A thin film of CuONPs deposited on silica glass plate by dropping few drops of the CuONPs solution on the plate and then allowed to dry at 30 °C for overnight. Images were taken, with AFM (Model Ntegra Prima AFM, NT-MDT, Russia).

### Biological characterization of *Phormidium sp.* derived CuONPs

#### In vitro antioxidant potential of CuONPs

The standard protocols were adopted for determining antioxidant activity like; 2, 2′ -azino-bis 3-ethyl benzthiazoline-6-sulfonic acid (ABTS)^84^, 2,2′-diphenyl-1-picrylhydrazyl (DPPH)^85^, hydrogen peroxide (H_2_O_2_)^86^ and superoxide radical (SOR) scavenging assays^87^. These methods are based on inhibition of free radical, which vary greatly according to the generation of free radical, its reproducibility, and the endpoint. Further, their potential was compared with standard ascorbic acid. Suspensions of CuONPs were sonicated to avoid NPs agglomeration (details given in ESI Section S1). The absorbance was measured spectrophotometrically against the corresponding blank solutions and the percentage inhibition of free radical scavenging was calculated using following equation^18^.

10 $${\text{\% Inhibition}}/{\text{Scavenging}} = \frac{Ac - At}{{Ac}} \times 100$$

where where Ac = Absorbance of control, At = Absorbance of test. The IC50 values of CuONPs were calculated and compared with the standard ascorbic acid.

#### Determination of antimicrobial and synergistic activity of CuONPs

To evaluate the antibacterial efficacy of biologically synthesized CuONPs, broth microdilution method as recommended by CLSI^88^ was performed against Gram-negative (*Escherichia coli, Klebsiella pneumonia*) as well as Gram-positive (*Bacillus cereus, Staphylococcus aureus*) bacteria. Antifungal activity was also determined against *Candida albicans* and *Candida glabrata* following standard guidelines of CLSI^89^. Different concentration of CuONPs (0.24–1000 µg ml^−1^), streptomycin and fluconazole as positive control, were placed into 96-well plate in a final volume of 100 µl. The test pathogens were harvested and their turbidity was assessed according to the McFarland 0.5 standard. Then, 100 µl of cell cultures (approximately 2.5 10^3^ cells per ml) were placed into the 96-well microtitre plate (Tarson) and incubated at 37 °C for 24 h. After incubation, the growth/turbidity was recorded at 600 nm using a spectrophotometer^18^. The lowest concentration of CuONPs at which no visible growth occurred represented its MIC (Minimum Inhibitory Concentration) value. All experiments were concurrently performed in triplicates.

The antimicrobial synergistic activities of CuONPs in combination with the standard antibiotic/fungicides were evaluated by the checkerboard assay^90^. A microtitre plate was inoculated with 50 µl of CuONPs (0.48–125 µg ml^−1^) and standard antibiotic/fungicides (0.24–31.2 µg ml^−1^). Each well was inoculated with 100 µl of microbial suspension to make up the final volume 200 µl and checkerboard plates were incubated overnight at 37 °C. The fractional inhibitory indexes (FIC) were calculated according to the Eq. (11).

11 $${\text{FIC}}_{{{\text{index}}}} = \frac{{\text{MIC of test sample in combination}}}{{\text{MIC of test sample alone}}}{ } + \frac{{{\text{MIC of antibiotic}}/{\text{antifungal in combination}}}}{{{\text{MIC of antibiotic}}/{\text{antifungal alone}}}}$$

where synergy and antagonism were defined by FICI ≤ and > 4, respectively. Synergy was defined by FICI < 0.5, partially synergistic were defined by 0.5 < FICI < 1, whereas indifferent was defined by FICI ≤ 4^91^.

#### Anti-inflammatory activity of biogenic CuONPs

Inhibition of albumin denaturation is used to analyze the anti-inflammatory activity of CuONPs by the modified method of Lavanya et al.^92^. The control solution (0.5 ml) consists of 0.45 ml of bovine serum albumin (1% w/v aqueous solution) and 0.05 ml of distilled water. The test sample (0.5 ml) consists of 0.45 ml of distilled water and 0.05 ml of CuONPs (25, 50, 75, 100, 125, 150 µg ml^−1^). The standard solution (0.5 ml) consists of 0.45 ml of bovine serum albumin (1% w/v aqueous solution) and 0.05 ml of various concentrations of diclofenac sodium. All the above solutions were adjusted to pH 6.3 using 1N HCl. Firstly, the samples were incubated at 37 °C for 20 min followed by the additional incubation at 57 °C for 5 min. After being cooled, 2.5 ml of phosphate buffer was added to the above prepared solutions. The absorbance was measured using a UV–Visible spectrophotometer at 416 nm. The control represents 100% protein denaturation and the results were compared with diclofenac sodium and percentage inhibition of protein denaturation was calculated using Eq. (12).

12 $${\text{\% Inhibition}} = 100 - \frac{{{\text{Optical density of control}} - {\text{optical density of test sample}}}}{{\text{Optical density of control}}} \times 100$$

#### Biocompatibility assay of CuONPs

Biocompatibility of CuONPs derived from *Phormidium* sp. was carried out quantitatively by 3-(4,5-dimethylthiazol-2-yl)-2,5-diphenyltetrazolium bromide (MTT) assay and qualitatively by 4′,6-diamidino-2-phenylindole (DAPI) staining using the method of Mossman^93^ and Liu et al.^94^. Briefly, 1 × 10^4^ ml^−1^ cell in their exponential growth phase were seeded in 96 well plates with Dulbecco’s modified eagle’s media (DMEM) along with 5% (v/v) Fetal Bovine Serum (FBS) and 1% antibiotics and antimycotic solution (Gibco). Incubated for 24 h at 37 °C in a humidification incubator with 5% CO_2_. After incubation the cells were treated with different conc. of CuONPs (20, 40 60, 80, 100, 120 and 140 µg ml^-1^), incubated for 24 h and washed with PBS thrice. After 24 h of incubation, in each well added MTT dye (5 mg/ml) was mixed with DMEM complete media in 9:1 ratio and again incubated at 37 °C for 3–4 h. Then, media was removed and 100 µl of DMSO was added in each well. The plates were analyzed in a microplate reader (BIO-RAD microplate reader-550) at 595 nm and percentage viability was calculated using Eq. (13).

13 $${\text{\% of viable cells}} = { }\frac{{{\text{A}}_{{{\text{T }} - }} {\text{ A}}_{{\text{B}}} }}{{{\text{A}}_{{\text{C}}} { } - {\text{ A}}_{{\text{B}}} }}{ } \times 100$$

where A_C_ = absorbance of the control: A_T_ = absorbance of the treated cells, A_B_ = absorbance of the blank. IC50 values of CuONPs were determined for each cell line.

Cells were stained with fluorescent 4',6-diamidino-2-phenylindole (DAPI) to detect nuclear condensation and blebbing. After 48 h of treatment, the cells in a 12 well plate were washed with PBS thrice. Cells were then fixed with 4% paraformaldehyde for 8 min and then washed with PBS thrice. After that, the cells were permeabilized by treating with 0.1% Triton X-100 for 2 min. Again washed 3 times with PBS and treated with fluorescent dye DAPI 1 μg/ml. After 5 min PBS was added to the well to keep the cells hydrated while imaging under fluorescent microscope ZOE Fluorescent Cell Imager, (Bio-Rad)^17^.

#### Photocatalytic degradation by CuONPs

The CuONPs derived from *Phormidium sp.* were also evaluated for degradation of methylene blue (MB) dye. For this 5, 10, 15, 20 and 25 mg/L of CuONPs were added to 100 ml of aqueous solution containing 25 mg/L of MB dye. Prior to the irradiation (500 W xenon lamp), the suspensions were magnetically stirred for 1 h in the dark to reach an adsorption/desorption equilibrium between CuONPs and the dye solution. The suspension mixtures were withdrawn at regular interval and centrifuged. In order to determine the remaining dye concentration the absorption spectra were recorded at 665 nm for (MB) using UV–Vis spectrophotometer^77^. Another set (control) was allowed to react under identical condition without CuONPs (photocatalyst). The percentage degradation efficiency for photocatalytic reaction was calculated using the Eq. (14).

14 $${\text{\% Degradation}} = \frac{{{\text{A}}_{{0}} - {\text{A}}}}{{{\text{A}}_{{0}} }} \times 100$$

where A_0_ and A are the initial and final absorbance of MB dye at λ_max_, respectively.

#### Identification of CuONPs induced free radical in dye degredation

Specific free radical scavenger protocols were adopted to identify their role in degradation of methylene blue by CuONPs. Different scavenger were added in the reaction mixture, viz. ammonium oxalate (h^+^ scavenger), p-benzoquinone (·O_2_^−^ scavenger) and tert-butanol (·OH scavenger)^95^. CuONPs (10 mg/L) and radical scavengers (10 mM) were placed into 100 ml of 25 mg/L dye solution along with respective scavengers. The reaction mixtures were placed under sunlight for 1 h. Finally the degradation rate of the dye was calculated to determine the active role of scavenger species.

### Statistical analysis

Data analysis was carried out using standard statistical software Origin 8.1. Descriptive statistical measures, especially the mean and standard deviation, were used to summarize the collection of data for each measurement. Two-way analysis of variance was used to evaluate the influence of independent variables as well as possible interactions between them in the antibacterial and antioxidant activity study. Values of *p < 0.05 were considered as statistically significant. Each test was performed in triplicates and results were presented as mean ± SD.

## Supplementary Information

Supplementary Information.

## Data Availability

The datasets generated during and/or analyzed during the present study are available from the corresponding author on reasonable request.
