# Supplementary material for: Toxicological assessment of Phormidium sp. derived copper oxide nanoparticles for its biomedical and environmental applications
Source: Sci Rep. 2023 Apr 17;13:6246. doi: 10.1038/s41598-023-33360-3 (PMC10110551; doi:10.1038/s41598-023-33360-3)

**Electronic supplementary information**

**Journal name:** Scientific report

# Toxicological assessment of *Phormidium sp.* derived copper oxide nanoparticles for its biomedical and environmental applications

Nida Asif[1], Rakhshan Ahmad[1], Samreen Fatima[1], Shehzadi Shehzadi[1], Tabassum Siddiqui[1], Almaz zaki[2] and Tasneem Fatma[1]*

[1]Department of Biosciences, Jamia Millia Islamia, New Delhi, India

[2]Department of Biotechnology, Jamia Millia Islamia, New Delhi, India

***Correspondence**

**Tasneem Fatma**

Professor

Department of Biosciences

Jamia Millia Islamia

Jamia Nagar, New Delhi-110025,

Phone no.: 9891408366

Email: fatma_cbl@yahoo.com

## Materials and methods

### Antioxidant activity of CuONPs

**ABTS scavenging assay:** The free radical scavenging activity of ZnO NPs was determined by ABTS (2, 2′ -azino-bis 3-ethyl benzthiazoline-6-sulfonic acid) diammonium salt and using ascorbic acid as a positive control, by using the modified protocol of Ree et al [21]. The ABTS radical cations were generated by mixing 7 mM ABTS solution with 2.45 mM potassium persulphate and reaction mixture was kept at room temperature for overnight in dark. To 0.2 ml of various concentrations of the ZnO NPs/standard, 1.0 ml of ABTS solution was added to make a final volume of 1.2 ml. Incubated at 37◦C for 20 min and absorbance was measured spectrophotometrically at 734 nm. The scavenging ability was calculated as above using the Eq. (1).

**DPPH scavenging assay:** The free radical scavenging activity of ZnO NPs was measured by 2,2′ -diphenyl-1-picrylhydrazyl (DPPH) method, by using the modified protocol of Muniyappan and Nagarajan [22]. To the 200 μl of 2, 2′ -diphenyl-1-picrylhydrazyl (DPPH) solution, 10 μl of each of the sample suspension or standard (ascorbic acid) solution was added. The mixture was shaken vigorously and allowed to stand for 30 min in dark. After 30 min, the reduction of the DPPH radical was determined by measuring the absorbance at 517 nm. The free radical scavenging activity was calculated as above using the Eq. (1).

**Hydrogen peroxide radical scavenging assay:** The decay or loss of hydrogen peroxide is measured spectrophotometrically by using the modified protocol of Smirnoff and Cumbes [23]. A solution of hydrogen peroxide (43 mM) was prepared in phosphate buffered saline (PBS, pH 7.4). Various concentrations of ZnO NPs or standard (ascorbic acid) in methanol (1 ml) was added to 2 ml of hydrogen peroxide solution in PBS. The reaction mixtures were incubated at room temperature for 10 min and the absorbance was measured at 230 nm. The percentage inhibition of H2O2 free radical scavenging was calculated as above using Eq. (1).

**Superoxide radical scavenging assay:** The assay was performed by using the modified protocol of Zhishen et al [24]. The reaction mixture of riboflavin ($3 \times 10^{-6}$ M), methionine ($1\times 10^{-2}$M) and nitroblue tetrazolium (NBT) ($1 \times 10^{-4}$M) were added in 0.05 M of potassium phosphate buffer to form a solution of 50 μM (pH 7.8). To 3 ml to the above reaction mixture, 300 μl of ZnO NPs

was added and kept in light for 30 min. A similar set was kept in dark for the same time as blank. The absorbance was measured at 560 nm and percentage scavenging of free radicals was calculated by using Eq. (1).

**Supplementary figures and tables:**

**Figure S1:** GC–MS profile of *Phormidium sp.* cell extract

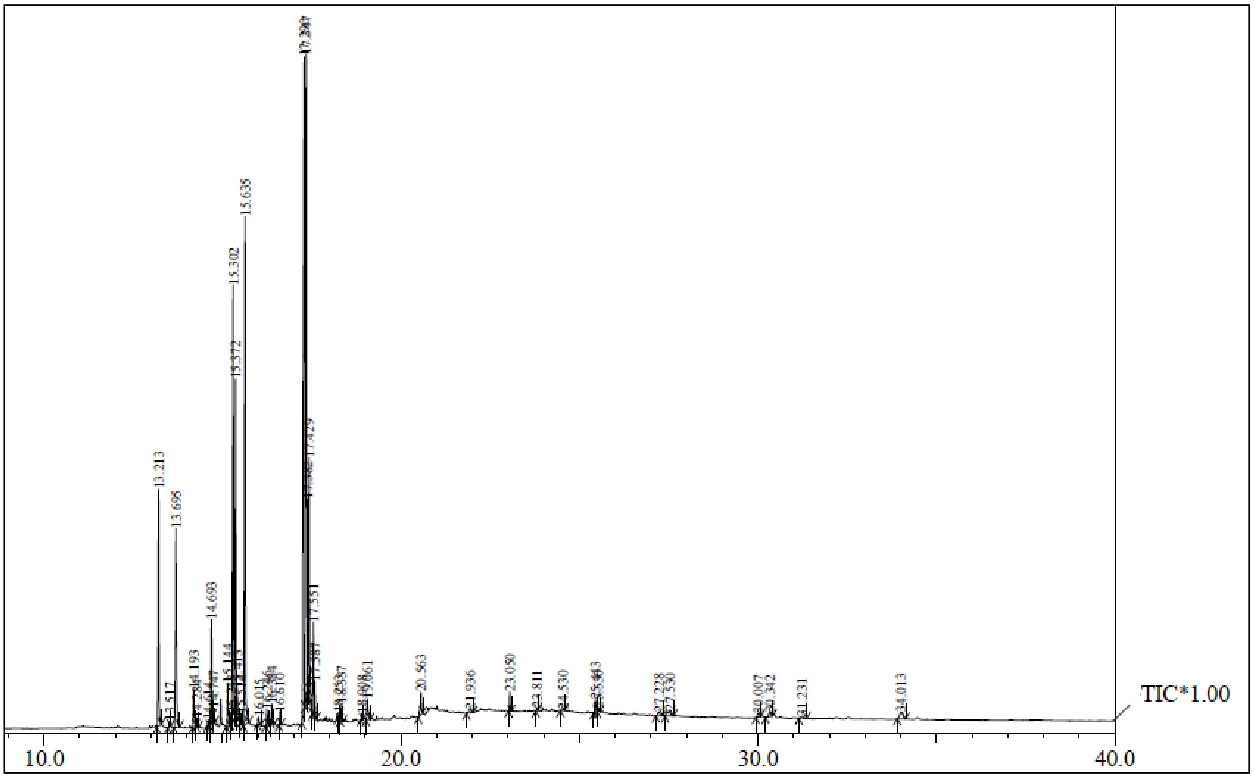

**Table S1:** List of compounds present in cell extract of *Phormidium sp.* and their activities identified by gas chromatography mass spectrometry (GC-MS) analysis.

| RT | Name of compound | Peak area% | Molecular formula | Compound nature | Biological activity |
|---|---|---|---|---|---|
| 13.213 | Nonadecane | 5.32 | $C_{19}H_{40}$ | Alkane hydrocarbon | Antimicrobial, antioxidant, anticancer |
| 13.695 | Heptadecane | 5.35 | $C_{18}H_{38}$ | Alkane hydrocarbon | Antioxidant, antimicrobial |
| 14.693 | Neophytadiene | 2.10 | $C_{20}H_{38}$ | Diterpine | Anti-inflammatory, anti-microbial |
| 15.302 | 9,12-Hexadecadienoic acid | 9.60 | $C_{17}H_{30}O_2$ | Palmitic acid | Antiandrogenic, antioxidant |
| 15.635 | Hexadecenoic acid | 12.7 | $C_{18}H_{36}O_2$ | Palmitic acid | Antibacterial |
| 17.290 | 9,12-Octadecadienoic acid | 21.09 | $C_{19}H_{34}O_2$ | Oleic acid | Anti-inflammatory, cancer preventive, flavor dermatitigenic |
| 17.347 | 9-Octadecenoic acid | 17.06 | $C_{19}H_{36}O_2$ | Fatty acid | Antimicrobial, acaricidal |
| 17.429 | Phytol | 4.78 | $C_{20}H_{40}O$ | Diterpene | Antimicrobial, anticancer, anti-inflammatory |
| 23.050 | Squalene | 0.39 | $C_{30}H_{50}$ | Triterpine | Antioxidant, antitumor, antibacterial |
| 25.443 | Tocopheryl methyl | 0.38 | $C_{29}H_{49}DO_2$ | Ester | Antioxidant |

**Figure S2:** Pictorial presentation of synthesis of CuONPs

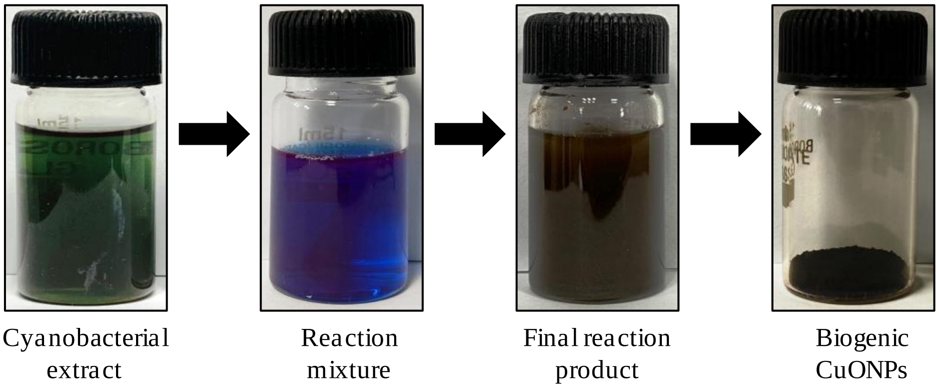

**Table S2:** Particles size and peak indexing of *Phormidium* derived CuONPs.

| 2θ of the intense peak (deg) | θ of the intense peak (deg) | FWHM of intense peak β in radian | Size of the particle (D) nm | Average size (nm) | d-spacing nm | (h k l) |
|---|---|---|---|---|---|---|
| 35.5 | 17.7 | 0.013 | 40.5 | **22.5 nm** | 0.253 | |
| 38.7 | 19.3 | 0.017 | 30.3 | | 0.232 | |
| 48.7 | 24.3 | 0.020 | 25.5 | | 0.187 | |
| 53.6 | 26.8 | 0.046 | 10.8 | | 0.171 | |
| 58.2 | 29.1 | 0.033 | 15.1 | | 0.157 | |
| 61.5 | 30.7 | 0.030 | 16.7 | | 0.150 | |

**Figure S3:** Anti-inflammatory activity of *Phormidium* derived CuONPs

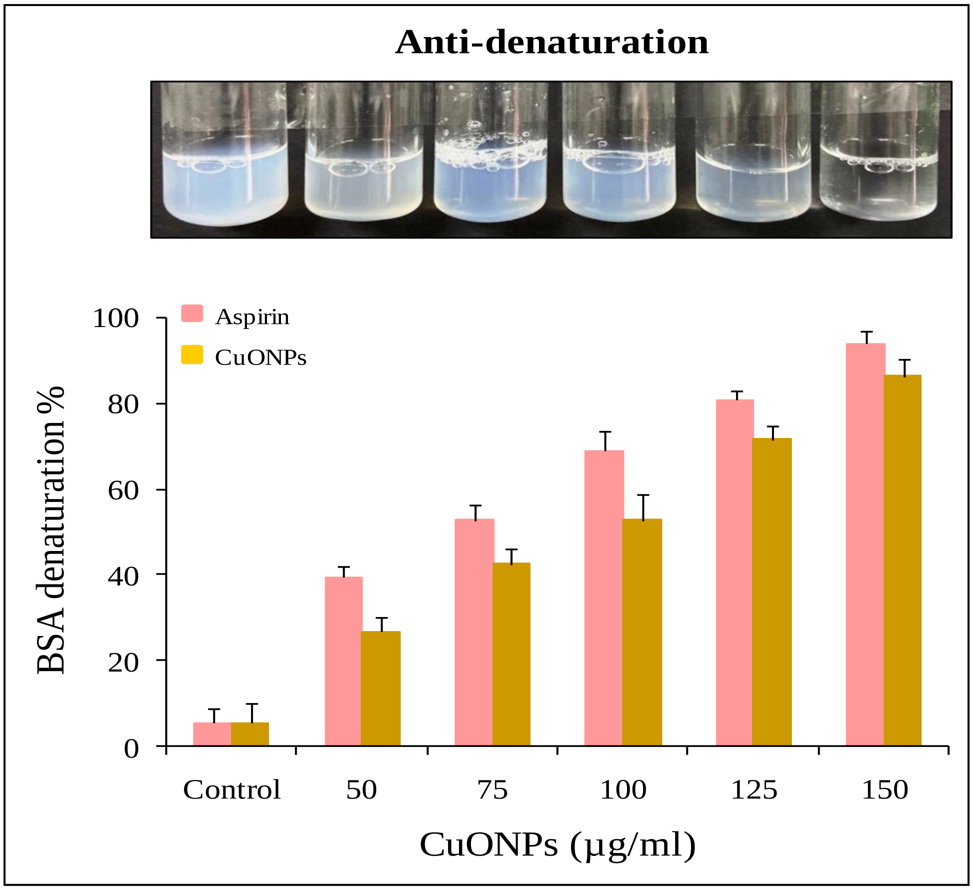

**Figure S4:** Degradation of methylene blue (MB) with CuONPs (10 mg/L) in the presence of scavengers i.e. ammonium oxalate (AO), *p*-benzoquinone (p-BQ) and *tert*-butanol (t-BuOH) with 60 min irradiation time and 25 mg/L of dye. Experiments were performed in triplicates; bars represent the mean of values and error bars represent mean ± SD (*P < 0.05).

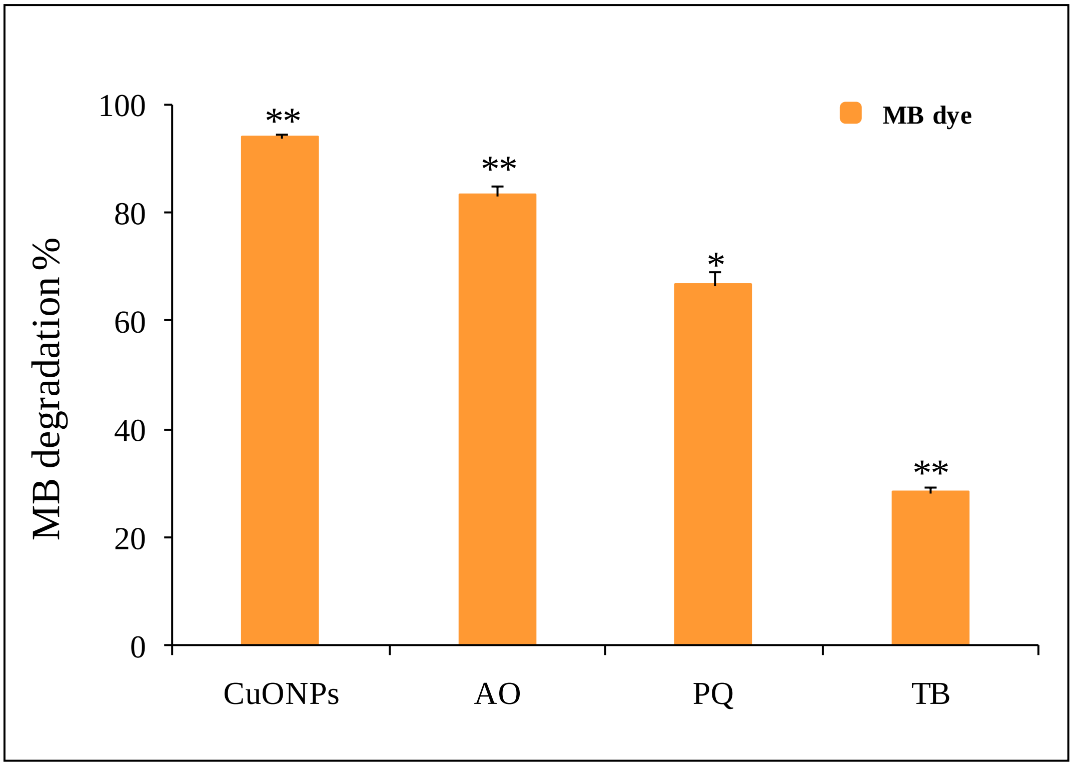

Supplement: Supplementary file 1 — Supplementary Information. [file 41598_2023_33360_MOESM1_ESM.pdf]
